# Evaluation of nutritional and elemental compositions of green and red cultivars of roselle: *Hibiscus sabdariffa* L.

**DOI:** 10.1038/s41598-020-80433-8

**Published:** 2021-01-13

**Authors:** Serifat Olatundun Salami, Anthony Jide Afolayan

**Affiliations:** 1 Medicinal Plants and Economic Development (MPED) Research Centre, Department of Botany, University of Fort Hare, Alice, 5700 Eastern Cape South Africa; 2 Department of Agricultural Education, Federal College of Education (Special), Oyo, PMB 1069 Oyo State Nigeria

**Keywords:** Conservation biology, Plant sciences

## Abstract

Roselle—*Hibiscus sabdariffa* L. leaves at different stages of growth, calyces and seeds were analyzed for nutritional and anti-nutritional factors. All the treatments contained a good but varied proportion of carbohydrate in a range of 26.93–54.13%, crude protein from 5.7 to 27.06%, crude fat 1.16–13.09%, crude fibre 15.75–36.10%, energy 631.36–1065 kJ, ash 6.08–13.74% and moisture content 6.00–9.7%. The vitamins A, C and E were also found to be present in all the treatments in a different but substantial amount. The calcium, magnesium and iron contents in all the treatments were higher than the recommended daily allowance of 1250, 350, and 15 mg for adults, while the phosphorus and sodium values in all the treatments were below 1000 and 1500 mg RDA for adults. However, the Na + /K + for all the treatments were lower than 1. The values of anti-nutrients in the samples were small except post-flowering red which had high phytate content of 21.02%, although this can be easily reduced during processes like boiling and cooking. Thus, both cultivars of roselle contain high nutritional, elemental, and vitamins compositions and small content of anti-nutrients.

## Introduction

The population is increasing at an alarming rate while forest resources and land available for agriculture are reducing daily, there is a need to put available resources into maximum use to meet the need of man^1^. Edible wild plants are important sources of dietary nutrients, which contribute to the proper growth and functioning of the body^2^. Food and Agriculture Organization (FAO) reported that billions of people in Africa depend on edible wild plants in their diets^3^. According to^4^, the global demand for food is expected to increase by 60% in the year 2050 because of increase in the population. Edible wild plants are important sources of dietary nutrients, which contribute to the proper growth, and functioning of the body^5^. To prevent hunger in the nearest future, more edible wild vegetables, which are rich in nutrients, must be incorporated into the diet of people, especially in Africa.

Roselle is one of the important vegetables consumed by man and livestock. Roselle tender shoots, succulent calyces and immature fruits are chopped and added to sauce; the younger herbages are sometimes eaten raw in salads^6^. The most exploited part of the plant is its calyces, which may be green, red or dark green^7,8^. Calyces of green roselle are commonly used to prepare soup, stew and sauces while red calyces are used to prepare relishing drink called roselle drink (English), Bissap (Senegal), Karkade (Saudi Arabia) or Zoborodo in Nigeria^9^. Roselle is highly regulated from the standpoint of nutritional, medicinal and other economic value. The plant is a cheap source of vegetable protein, fat and minerals^10^*.* Ref.^8^ affirmed that the calyces of roselle are very rich in vitamins, riboflavin with some major minerals present. Also^11^, reported that calyces contained 9X vitamin C than *Citrus sinensis*. The oil extracted from the seeds is used for cooking, soap making, and cosmetics while the residue is used for feeding chicken and livestock^12^. In Nigeria, ‘kuiru’ an important ingredient for the traditional seasoning ‘iru’ is derived from the seeds. The plant has also been reported in various researches to contain more essential elements^12^. In addition to its use as food, various parts of roselle have been utilized in traditional medicine for the prevention of diseases such as diabetics, cancer, hypertension and obesity^13^. Regular consumption of roselle may reduce nutritional deficiency problems such as night blindness, scurvy and rickets^14^. However, it has been reported that roselle contains some anti-nutritional factors, which prevent the utilization of some nutrients in the body, these include oxalate, phytate and saponin. Anti-nutrients are chemicals, which have been evolved by plants for their defence (among other biological functions). These chemical substances (antinutrients) in the plants are advantageous to man and animal health if consumed at the appropriate amount but hazardous at high quantity. Anti-nutrients have been shown to reduce blood glucose and insulin responses to starchy foods and /or cholesterols^15^. Besides, phytate, tannins, saponins, protease inhibitor and oxalate have been associated with reduced cancer risks.

At relatively high amount, they reduce the maximum utilization of nutrients especially proteins, vitamins and minerals and thus prevent the optimal exploitation of the nutrients present in food and decreases the food nutritive values^15^. High quantity of phytate, for example, prevents the absorption of Calcium in the body. In many developing countries, roselle nutritional benefits have not been fully exploited despite the existing potential for wider applications in food industries. Moreover, people consume red cultivars while green cultivars are neglected. In addition, previous works on roselle targeted calyces while leaves were not much investigated. Although the leaves are consumed for vegetable and salad, the stage of growth at which the plant can be harvested for maximal nutritional benefits and anti-nutrient compositions at various growth stages is limited in research. Therefore, this study aims to determine and compare the nutritional composition of leaves, calyces and seeds of red and green roselle, investigate and compare the anti-nutrient contents of green and red roselle and examine variability in the nutritional contents of the cultivars at various stages of growth.

## Results

### Effects of the treatments on the proximate compositions

In this study, variations occurred among some treatments. The values of the carbohydrate content in the samples ranged from 26.93 ± 0.7 to 54.15 ± 0.18%. The post-flowering stage had the highest carbohydrate content, followed by the carbohydrate content in the seeds, the lowest carbohydrate value was recorded in the calyces of red roselle. There was, however, no significant difference (P < 0.05) in the carbohydrate content of leaves of both cultivars at pre-flowering and post-flowering stages as well as seeds of both cultivars. Moisture content in the samples ranged from 6.00 ± 0.27 to 9.7 ± 0.53%, the values of moisture in the leaves, calyces and seeds of green roselle being generally higher than red. Calyces had the highest moisture content followed by seeds and leaves.

Ash content in the samples, however, ranged from 6.08 ± 0.05 to 13.74 ± 0.21%, the ash content in the leaves, calyces and seeds of red roselle was significantly (P < 0.05) higher than green. The least value was recorded in roselle seeds. The protein content ranged from 5.7 ± 0.6 to 27.06 ± 0.2%. Green roselle seeds had the highest protein content, although not significantly higher than the content in the seeds of red roselle (22.5 ± 0.60%), followed by pre-flowering red (21.28 ± 0.60%). The protein content recorded in the green roselle leaves at all stages of growth was at par statistically. Red calyces had the least content of protein.

The crude fibre content in the sample ranged from 15.75 ± 0.01 to 36.10 ± 0.82%. Seeds of green roselle had the highest crude fibre content while red calyces had the least crude fibre content. No significant variation observed on the leaves during pre-flowering and flowering stages as well as in the calyces and seeds of both cultivars. Crude fat content in the samples ranged from 1.16 ± 0.1 to 13.09 ± 0.29%. The fat contents in the leaves of red cultivar were generally higher than those in green, however, green calyx was higher than red. The fat contents in the seeds were the highest.

The energy values in the samples were in the range of 631.36 ± 1.10 to 1065.24 ± 4.87 kJ. Energy values of the leaves, calyces and seeds were at par for both cultivars (Table 1). Similarly, Table 2 showed that there was no significant difference (P < 0.05) in the nutrient compositions of roselle plant parts either seed, calyces or leaves. Also, at all stages of growth, no variation occurred in the nutrient compositions of roselle except in the crude fat content which varied significantly (P < 0.05). Although there were differences in the moisture, ash and crude fat compositions of the two cultivars, there was no significant difference in the carbohydrate, protein, crude fibre and energy compositions of the cultivars.

**Table 1:** Proximate contents of samples of green and red roselle.

Treatments	Carbohydrate %)	Moisture (%)	Ash (%)	Crude protein (%)	Crude fibre (%)	Crude fat (%)	Energy (KJ)
PrG	38.56 ± 1.09^c^	7.11 ± 0.07^de^	11.01 ± 0.26^c^	17.27 ± 0.90^c^	24.5 ± 1.13^c^	3.72 ± 0.32^a^	920.8 ± 20.83^b^
PrR	37.24 ± 0.9^c^	6.95 ± 0.13^de^	12.26 ± 0.08^b^	21.28 ± 0.60^b^	22.8 ± 3.00^c^	2.9 ± 0.042^b^	920.8 ± 14.48^b^
FG	48.33 ± 1.05^b^	7.51 ± 0.25^ cd^	10.45 ± 0.07^d^	17.11 ± 0.18^c^	30.7 ± 1.23^b^	1.65 ± 0.12^d^	724.35 ± 18.44^c^
FR	37.13 ± 2.72^c^	6.6 ± 0.05^ef^	13.74 ± 0.21^a^	16.95 ± 0.3^d^	25.04 ± 0.33^b^	2.55 ± 0.24^bc^	875.53 ± 0.40^c^
PoFG	47.74 ± 0.71^a^	6.33 ± 0.311^fg^	11.05 ± 0.02^c^	17.14 ± 0.21^c^	28.62 ± 0.71^b^	1.98 ± 0.05^d^	731.21 ± 0.90^d^
PoFR	54.15 ± 0.18^a^	6.00 ± 0.27^g^	10.8 ± 0.16^cd^	16.36 ± 0.13^d^	35.01 ± 0.83^a^	2.10 ± 0^c^	631.36 ± 1.10^e^
CG	39.65 ± 2.02^c^	9.7 ± 0.53^a^	9.35 ± 0.33^e^	15.30 ± 0.25^e^	17.52 ± 0.70^e^	1.74 ± 0.01^d^	890.06 ± 39.18^c^
CR	26.93 ± 0.71^d^	8.9 ± 0.07^b^	10.9 ± 0.32^cd^	5.7 ± 0.61^f^	15.75 ± 0.01^e^	1.16 ± 0.10^e^	911.67 ± 9.98^c^
SG	49.41 ± 0.55^a^	8.2 ± 0.26^c^	6.08 ± 0.05^f^	27.06 ± 0.21^a^	36.10 ± 0.82^a^	13.09 ± 0.29^a^	1065.24 ± 4.866^a^
SR	53.82 ± 1.04^a^	7.82 ± 0.25^c^	6.13 ± 0.06^f^	22.5 ± 0.69^a^	34.65 ± 1.35^a^	11.53 ± 5.23^a^	1018.89 ± 14.21^a^

Values shown are mean ± SD; different letters along a column represent significant differences at p < 0.05 among all the treatments.

*PrG* pre-flowering green, *PrR* pre-flowering red, *FG* flowering green, *FR* flowering red, *PoFG* post-flowering green, *PoFR* post-flowering red, *CG *calyces green, CR calyces red, *SG* seed green, *SR* seed red.

**Table 2:** ANOVA analysis of nutritional components of roselle.

P-values from two-way ANOVA							
	Carbohydrate (%)	Moisture (%)	Ash (%)	Crude protein (%)	Crude fibre (%)	Crude fat (%)	Energy (KJ)
Plant parts	0.000	0.000	0.000	0.000	0.000	0.000	0.000
Stages of growth	0.000	0.001	0.000	0.000	0.000	0.503	0.000
Cultivars	0.023	0.275	0.461	0.000	0.044	0.810	0.023
Plant parts × cultivars	0.001	0.036	0.000	0.000	0.001	0.686	0.001
Stages of growth × cultivars	0.064	0.012	0.000	0.000	0.002	0.888	0.064

Also, the interaction between plant parts and cultivars gave no variation in the nutrient compositions of all treatments except fat content which varied significantly. Furthermore, no significant variation a(P < 0.05) in the interaction of stages of harvest and cultivars on moisture, ash, crude protein and crude fibre compositions of roselle, however, significant differences in their interaction occurred on carbohydrates, crude fat and energy contents.

### Effects of the treatments on mineral contents

The magnesium content in the samples ranged from 365 ± 7.07 to 555 ± 21.2 mg. The value of magnesium in red roselle at the pre-flowering stage was the highest, followed by that of green calyces then red calyces. Post-flowering red had the least value. Calcium content in the samples varied from 245 ± 7.07 to 2375 ± 35.36 mg. Ca content in red post-flowering was higher than other treatments, followed by pre-flowering red (1820 ± 14.14 mg). Seeds of red roselle had the least value of Ca though not significantly lower than green seeds (270 ± 0 mg). Red calyces Ca content was higher than the value in green calyces.

Sodium (Na) content in the samples ranged from 10.0 ± 0 to 40 ± 28.28 mg. Red roselle seeds had the lowest Na content while red calyces had the highest value. Na content in the leaves and calyces were higher than those in the seeds, although the value in green was higher than red. Manganese (Mn) content in all the treatments ranged from 4.05 ± 0.07 to 29.4 ± 0.42 mg. Red calyces Mn content was the highest while the lowest value was recorded for flowering green. Mn values for both cultivars at the pre-flowering stage and red at the flowering stage, as well as green calyces, were not significantly different. Also, the Mn value at flowering and post-flowering stages of green roselle, as well as seed of red cultivar, were not different.

Zinc (Zn) content in the samples ranged from 2.3 ± 0 to 5.55 ± 0.07 mg. The highest value was recorded for red seeds followed by green seeds (5.0 ± 0.14 mg); post-flowering red had the least value. There was no much variation in the content of Zn in roselle. Phosphorus content ranged from 125 ± 7.07 to 645 ± 7.07 mg. The phosphorus content in the seeds of green roselle was higher than other treatments while red seeds and red calyces had the same but least value. The P content at pre-flowering and flowering stages were not significantly different.

Copper (Cu) content in all the treatments varied from 0.5 ± 0 to 1.1 ± 0 mg. The Cu content in the seeds of red roselle was the highest and varied significantly from others including green seeds. The values of Cu in the two cultivars varied significantly in leaves (at each growth stage), calyces and seeds. Iron (Fe) was in the range of 15.31 ± 1.41–111.85 ± 12.8 mg, flowering red had the highest Fe content while flowering green had the least. There was no significant difference between the values at the pre-flowering stage of red and green roselle 26.25 ± 3.0 mg and 27.62 ± 1.13 mg as well as the Fe value at the post-flowering stage of red roselle. While the value at the post-flowering stage of green roselle did not vary significantly (P < 0.05) with the values in the calyces of both roselle.

Potassium (K) was high in all the samples having a range of 1415 ± 7.07–2630 ± 127.28 mg. Pre -flowering red had the highest content of K followed by post-flowering green 2405 ± 77.80 mg while red seeds had the least content. Green calyces and pre-flowering green leaves were not significantly different while K content in red calyces was not significantly different from the values at the flowering stage of both cultivars. Also, K value at the post-flowering stage of red did not vary significantly from the values in the seeds of both cultivars. Sodium Potassium ratio (Na + /P +) for all the samples was far less than one, the highest value being 0.018 while the lowest was 0.01 (Table 3).

**Table 3:** Elemental composition of green and red roselle.

		Composition (mg/100 g)								
Treatments	Mg	Ca	Na	Mn	Zn	P	Cu	Fe	K%	Na + /K +
PrG	385 ± 7.07d	1555 ± 63.63d	40 ± 14.14a	6.35 ± 0.4b	3.85 ± 0.07bc	395 ± 7.07b	0.7 ± 0c	27.6 ± 1.13 cd	2305 ± 120.21bc	0.017
PrR	555 ± 21.2a	1820 ± 14.14b	35 ± 7.07ab	6.5 ± 0.9b	3.55 ± 0.212d	355 ± 7.07bc	0.8 ± 0b	26.25 ± 3.606 cd	2630 ± 127.28a	0.013
F G	465 ± 7.07b	1785 ± 49.5b	35 ± 7.07ab	4.05 ± 0.07d	3.5 ± d	390 ± 0b	0.8 ± 0b	15.31 ± 1.41e	2175 ± 7.07c	0.016
FR	415 ± 7.07 cd	1770 ± 42.42	40.0a	6.3 ± 0.07b	2.65 ± 0.07e	280 ± 14.14d	0.7 ± 0c	111.85 ± 12.8a	2225 ± 148.50c	0.018
PoFG	465 ± 7.07b	1750 ± 28.28bc	25 ± 7.07ab	4.15 ± 0.07d	3.15 ± 0.07d	330 ± 0c	0.8 ± 0b	17.3 ± 0de	2405 ± 77.80b	0.01
PoFR	365 ± 7.07d	2375 ± 35.36a	20 ± 14.14ab	5.2 ± 0.14c	2.3 ± 0e	220 ± 0e	0.55 ± 0.07e	27.65 ± 1.2 cd	1455 ± 21.21d	0.013
C G	510 ± 7.07a	1110 ± 70.7e	30 ± 14.14a	6.45 ± 0.64b	3.95 ± 0.35b	370 ± 49.5bc	0.85 ± 0.07ab	17.95 ± 0.8de	2350 ± 28.28bc	0.013
CR	440 ± 14.14c	1675 ± 70.7c	40 ± 28.28a	29.4 ± 0.42a	4 ± 0.42b	125 ± 7.07f	0.5 ± 0d	21.2 ± 2.97de	2265 ± 63.63c	0.013
SG	370 ± 0d	270 ± 0f	15 ± 7.07ab	5.7 ± 0.14c	5.0 ± 0.14a	645 ± 7.07a	0.9 ± 0ab	36.1 ± 1.00bc	1565 ± 21.21d	0.01
SR	395 ± 7.07d	245 ± 70.7f	10.0b	4.35 ± 0.07d	5.55 ± 0.07a	125 ± 7.07f	1.1 ± 0a	46.2 ± 7.64b	1415 ± 7.07d	0.006

Values shown are mean ± SD; different letters along a column represent significant differences at p < 0.05 among all the treatments.

*PrG* pre-flowering green, *PrR* pre-flowering red, *FG* flowering green, *FR* flowering red, *PoFG* post-flowering green, *PoFR* post-flowering red, *CG* calyces green, *CR* calyces red, *SG* seed green, *SR* seed red.

Table 4 showed that plant parts and stages of harvest had no significant effects on the mineral composition of roselle except Na which showed a significant variation (P < 0.05). Also, the effects of cultivars on Na and copper were significant although no significant variation occurred in all other nutrient elements. Interaction of plant parts and cultivars had no significant effects on the elemental composition of roselle except Mg and Na which varied significantly. Also, the interaction of the stage of harvest with cultivars had no effects on the composition of nutrient elements except Na.

**Table 4:** ANOVA analysis of the elemental composition of roselle.

P-values from two-way ANOVA									
	Mg	Ca	Na	Mn	Zn	P	Cu	Fe	K
Plant parts	0.001	0.000	0.085	0.000	0.000	0.000	0.000	0.000	0.000
Stages of growth	0.030	0.000	0.203	0.000	0.000	0.000	0.010	0.000	0.000
Cultivars	0.002	0.020	0.731	0.000	0.069	0.000	0.188	0.000	0.000
Plant parts × cultivars	0.141	0.000	0.703	0.000	0.026	0.000	0.021	0.043	0.000
Stage of growth × cultivars	0.000	0.000	1.000	0.000	0.000	0.000	0.000	0.000	0.000

### Effects of the treatments on the vitamin content

Vitamin A content in the sample ranged from 131.51 ± 0 to 1236.15 ± 6.89 µg RAE/100 g DW. Pre-flowering green had a higher content of vitamin A, the value was, however, not significantly higher than that in pre-flowering red (1228.0 ± 25.0 µg RAE DW/100 g). Red calyces had the least value. The vitamin A values of both red seeds were higher than their calyces. At a concentration of 1 mg/ml (highest concentration), vitamin A standard (Retinol) absorbance value 0.2410 was lower than the values of the sample at the pre-flowering stage of green and red roselle (3.8036 and 3.7778 respectively), and almost equal to values at flowering stage of red (2.6713) and greater than values of other treatments. The higher the value of absorbance the higher the vitamin content when extrapolated from the standard curve. Hence, roselle at the pre-flowering stage a better source of vitamin A.

Vitamin C content in the samples ranged from 1.93 ± 0.02 to 4.49 ± 0.12 mg ascorbic acid/100 g DW. Although the value in red calyces was the highest, vitamin C contents in both calyces and leaves were higher than the values in the seeds. At the highest concentration (1 mg/ml), vitamin C absorbance value for ascorbic acid—standard (0.2487) was lower than the absorbance values of all the extracts which were all more than 1.000. All the treatments were a better source of vitamin C than ascorbic acid.

Vitamin E content in the treatments ranged from 35.11 ± 0.04 to 37.30 ± 0.03 mgα tocopherol/100 g DW. Although Vitamin E content in post-flowering red was higher, all other treatments had equal values (Table 3a). The value of vitamin E standard at a concentration of 1 mg/ml (3.7553) was far higher than the values in all the treatments which ranged from 0.5947 in post-flowering red to 0.1907 in pre-flowering green. Roselle contains low vitamin E value (Table 5).

**Table 5:** Vitamins contents of samples of green and red roselle.

Treatment	Vitamin A (µg retinol/100 g DW)	Vitamin C (mg ascorbic acid/100 g DW)	Vitamin E (mg α-tocopherol/100 g DW)
PrG	1236.15 6.89^a^	2.55 ± 0.10^bc^	35.11 ± 0.04^b^
PrR	1228.0 ± 5.42^a^	4.47 ± 0.24^a^	35.90 ± 0.05^b^
FG	387.39 ± 14.9^c^	3.93 ± 0.10^b^	35.54 ± 0.04^b^
FR	886.07 ± 36.07^b^	4.23 ± 0.04^a^	36.21 ± 0.01^b^
PoFG	303.92 ± 6.65^d^	4.15 ± 0.03^a^	35.57 ± 0.05^b^
PoFR	328.75 ± 10.5^cd^	3.96 ± 0.23^b^	37.30 ± 0.03^a^
CG	165 ± 4.05^f^	4.47 ± 0.24^a^	35.48 ± 0.03^b^
CR	131.51 ± 6.92^g^	4.49 ± 0.12^a^	35.64 ± 0.02^b^
SG SR	193.03 ± 4.03^e^ 160.57 ± 3.22^f^	1.93 ± 0.02^cd^ 2.11 ± 0.17^c^	35.34 ± 0.02^b^ 35.61 ± 0.01^b^

Values shown are mean ± SD; different letters along a column represent significant differences at p < 0.05 among all the treatments.

*PrG* pre-flowering green, *PrR* pre-flowering red, *FG* flowering green, *FR* flowering red, *PoFG* post-flowering green, *PoFR* post-flowering red, *CG* calyces green, *CR *calyces red, *SG* seed green, *SR* seed red.

Table 6 shows that plant parts, stages of harvest and cultivars had no significant effect on the composition of the vitamins. The interaction of plant parts with cultivars had significant effects on vitamins A and C contents; although the combination had no effects on vitamin E content. The interaction of the stage of harvest and cultivars had no effects on the content of the vitamins.

**Table 6:** ANOVA analysis of Vitamins in roselle.

P-values from two-way ANOVA				
	Vitamin A	Vitamin C	Vitamin E	
Plant parts	0.000	0.000	0.000	
Stages of growth	0.000	0.000	0.000	
Cultivars	0.000	0.016	0.022	
Plant parts × cultivars	0.093	0.783	0.000	
Stage of growth × cultivars	0.000	0.013	0.000	

### Effects of the treatments on anti-nutrient content

Alkaloids contents in the treatments ranged from 1.40 ± 0.13 to 3.9 ± 1.0%. Pre-flowering stage of both roselles had the highest values, red calyces and seeds had the lowest values while other treatments had equal values. Oxalate values in the sample ranged from 0.22 ± 0 to 0.92 ± 0.05%, the highest value was recorded at pre-flowering stage of green roselle, though not much varied from the contents in the calyces and pre-flowering stage of red roselle. Oxalate contents at the pre-flowering stage of roselle were higher than at the post-flowering, the seeds had the lowest value. The oxalate contents in roselle were generally low.

Phytate contents in the samples ranged from 0.60 ± 0.10 to 21.02 ± 0.82%. Phytate content in leaves of red roselle at flowering and post-flowering stages, as well as red calyces, were the same but higher than other treatments. Saponin content in the sample ranged from 3.35 ± 0.13 to 37.8 ± 9.19%. The result showed that flowering green had highest values than other treatments. The values of saponin in all other treatments were not significantly different from one another. Both seeds had equal but the lowest value of 3.35% (Table 7).

**Table 7:** Anti-nutrients contents of samples of green and red roselle.

Treatments	Alkaloids%	Oxalate%	Phytate%	Saponin%
PrG	3.44 ± 0.58^a^	0.92 ± 0.05^a^	3.10 ± 0.20^c^	3.9 ± 0.43^b^
PrR	3.90 ± 1.00^a^	0.72 ± 0.03^ab^	14.52 ± 0.94^b^	4.43 ± 0.64^b^
FG	2.30 ± 0.69^abc^	0.60 ± 0.1^b^	2.90 ± 0.27^c^	37.8 ± 9.19^a^
FR	2.70 ± 0.62^abc^	0.90 ± 0.2^a^	18.10 ± 0.40^b^	4.63 ± 0.64^b^
PoFG	2.80 ± 10.00^abc^	0.60 ± 0.1^b^	0.60 ± 0.10^d^	5.22 ± 2.2^b^
PoFR	2.70 ± 0.81^abc^	0.70 ± 0.2^b^	21.02 ± 0.82^a^	4.6 ± 1.22^b^
C G	2.24 ± 1.30^abc^	0.60 ± 0.1^b^	2.51 ± 0.31^c^	3.4 ± 1.72^b^
C R	1.90 ± 0.54^bc^	0.91 ± 0.03^a^	18.57 ± 0.06^b^	7.1 ± 0.27^b^
SG	2.61 ± 0.93^abc^	0.22 ± 0^c^	2.39 ± 0.13^c^	3.35 ± 0.53^b^
SR	1.40 ± 0.13^bc^	0.30 ± 0.04^c^	0.90 ± 0.08^d^	3.35 ± 0.13^b^

Values shown are mean ± SD; different letters along a column represent significant differences at p < 0.05 among all the treatments.

*PrG* pre-flowering green, *PrR* pre-flowering red, *FG* flowering green, *FR* flowering red, *PoFG *post-flowering green, *PoFR* post-flowering red, *CG* calyces green, *CR *calyces red, *SG* seed green, *SR* seed red.

ANOVA result shows that there was no significant difference in the alkaloid, oxalate, phytate and saponin contents on the leaves, calyces and seeds of green or red roselle at different stages of growth. Also, the interaction of plant parts and cultivars, as well as interaction of stages of growth and cultivars, showed no significant difference (Table 8).

**Table 8:** ANOVA analysis of antinutrient composition of roselle.

P-values from two-way ANOVA				
	Alkaloid (%)	Oxalate (%)	Phytate (%)	Saponin (%)
Plant parts	0.000	0.000	0.000	0.000
Stages of growth	0.000	0.000	0.000	0.000
Cultivars	0.000	0.000	0.000	0.000
Plant parts × cultivars	0.000	0.000	0.000	0.000
Stage of growth × cultivars	0.000	0.000	0.000	0.000

## Discussion

The proximate, elemental, vitamins and anti-nutrient compositions of roselle leaves (at different stages of growth), seeds and calyces varied in this study. The protein values observed in this study agrees with the reports of^16^ but lower than the value recorded by^17^ for roselle. In the present study, most of the treatments fell within protein representation of most legumes (17–30%) The study further indicates that leaves (at any stage of growth), calyces or seeds of either green or red roselle possessed the same composition of protein. The plant is thus a good source of protein^18^. This is contrary to^19^ who reported many variations in the protein composition of roselle seeds and calyces.

Also, the stages of harvest did not have any effect on protein components of roselle leaves. In other words, whether roselle is harvested at pre-flowering, flowering or post-flowering (mature) stage, the protein composition is the same. This result is contrary to the report of Attah et al. (2013) which affirmed Ecoytpe A7 of roselle leaves protein content decreased significantly between vegetative stage and flowering and then remained relatively constant up to maturity.

Dietary fat adds to the palatability of food through absorption and retaining of flavour^20^. Stages of harvest and cultivars influenced the fat content in the plant; as the crude fat content in green reduced from the pre-flowering stage and then increased from flowering to maturity, red roselle fat content reduced from young age to maturity. Thus, red roselle is better harvested at vegetative stage and green at post-flowering for high-fat content. Interaction of plant parts and cultivars influenced fat content in roselle. The fat content in the seeds (13.09 and 11.53% for green and red cultivar respectively) was higher than those in leaves and calyces and even higher than 8.5% reported for most vegetables. The values for most treatments in this study agree with those reported by^21^ for roselle calyces (2.2%). Lowest fat content in calyces may be the reason for roselle drink consumption by obese people. Carbohydrate content had the highest value of all the food components in roselle (in the range of 26.93–54.14%), leaves at post-flowering stage having higher carbohydrate value^22^. Opined that *Amaranthus caudatus* had higher carbohydrate content compared to other food nutrients. However^21^, reported higher values than the result of the present study 79.68% and 66.3% respectively for roselle. The difference may be genetic, or due to different areas of production or post-harvest handling. Carbohydrate is a rich source of energy. In this study, any part of roselle at any age whether green or red did not affect the carbohydrate content of roselle. Also, the interaction of cultivar and plant parts did not influence carbohydrate contents of roselle. However, the interaction of stages of growth and cultivar had much influence on the content of carbohydrate, hence, green roselle at flowering and post-flowering stages had more carbohydrate content than red roselle.

Ash content is an index of mineral nutrient in food substances^23^. Red calyces were found to contain higher ash content than green while green leaves ash content varies from red. Ash content in this study was higher than the values reported by^24^ (5.55%) and 5.98% reported for most leafy vegetables, however, the result fell within the value obtained by^25^ for *Solanum nigrum* (11.14–12.42%). This is a pointer to the fact that roselle is a depot of mineral nutrients. Findings in this study also showed that either calyx, seed or leaves of roselle at any age, has the same composition of ash. This contradicts the reports of^26^ which affirmed that leaves of roselle had higher ash content than calyces. Green roselle content of ash, however higher than red.

Crude fibre values recorded for all the treatments were exceptionally higher than those reported by^24^ (6.33%) for roselle seeds and^27^ (4.15–6.75%) and Luvonga (2012) for calyces (14.6%). Green calyces were found to be a better source of fibre than red while seeds were observed to be a better source of fibre in roselle. The high fibre content in vegetables serves as a serum cholesterol reducing agent and thus helps to prevent the risk of coronary heart diseases and hypertension^28^. Noteworthy, any cultivar whether red or green can be consumed to give same content of crude fibre in roselle.

The keeping quality of any food substances is a function of its moisture content. In this work, the moisture content values in all the treatments were not up to 10% recommended for most agricultural products; this implies that the roselle may have long kept time and low infestation by microbes in store^29^. Green roselle had more moisture content than red in this work, hence may have shorter keeping quality compared to red. The study found that any part of roselle at any age contains the same moisture content as others.

About 1–2% of calories of energy is enough for better living^30^. The high total energy content in roselle may be due to high carbohydrate and crude fat in some of these treatments. The energy content in roselle was independent of the part of the plant or cultivar of roselle. The combination of stages of growth with cultivar, however, influenced energy content. Thus, during flowering, the energy content in red roselle was lower than green and vice versa at the post-flowering stage. Energy values for both cultivars were the same when young but increased as they tend to maturity. This conforms with^22^ who affirmed that changes in the stage of growth bring changes in the energy content of *Amaranthus caudatus*.

Mineral nutrients are very essential in our bodies hence called essential nutrients which could be macro – those required in a greater amount than 100 mg per day and micro – those required in less than 100 mg per day^31^. Most of the treatments contained calcium, which was higher than 1200 mg recommended daily allowance (RDA). Ca is good for strong teeth and bones, lack of calcium results in a breakdown of bones. In this study, it was found that Ca content in roselle were not affected by parts of plants, cultivar and stages of growth. The interaction of cultivar with either plant parts or stages of growth did not affect Ca content.

Potassium content in all the treatments was the highest of all the mineral elements having a range of 1415–2630 mg/100 g. This confirms previous reports on roselle having higher K value than other nutrient elements. Potassium is essential for the electrolytic processes within the body. It is vital for the nervous system functioning, maintenance of body fluid, maintenance of correct rhythm of the heartbeat, clotting of blood and for contractile movement of the body. Roselle leaves, calyces and seeds may thus be useful for good physiological processes in the body. The RDA for potassium in adults is 4700 mg^32^, the plant can supply 50% of RDA if included in the diets of people. In this study, it was found that K content in roselle was not affected by parts of plants, cultivar and stages of growth. The interaction of cultivar with either plant parts or stages of growth also did not affect K content.

Magnesium is important in the transmission of nerve impulse and muscle contraction. The values of Mg recorded in this work for all the treatments were higher than 350 mg Recommended Daily Intake (RDI) for adults^33^. Roselle leaves, calyces and seeds may be added to the human diet for good nerve transmission. The content of Mg in roselle was not influenced by plant parts, cultivar or stages of growth. The interaction of plant parts and cultivars, however, influenced Mg in roselle. This contradicts the reports of^26^ which stated that calyces of roselle was higher in mineral content than leaves. Magnesium content was lower in seeds than other parts and had higher content in red seeds than green seeds while having higher content in green calyces than red calyces.

Copper is necessary for the proper growth, development and maintenance of bones, connective tissue, brain and heart. The values of copper in most of the treatments were higher than RDA 0.7 for children. The value obtained for the seeds was equivalent to RDA required by an adult (1.1 mg), all other treatments can supply more than 50% of this if roselle is taken as food. This study found that either plant part, stages of growth or their interactions did not affect the Cu content in roselle. The copper content in roselle was, however, affected by cultivar; red seed having higher Cu value than green while pre-flowering red was higher than pre-flowering green.

Iron is very important in blood formation. All the treatments were found to contain Fe higher than the RDI required (15 mg)^33^. Therefore, for blood enrichment in our body, the plant may be included in the diet. In this study, it was found that Fe content in roselle was not affected by parts of plants, cultivar and stages of growth. The interaction of cultivar with either plant parts or stages of growth also did not affect Fe content.

Phosphorus is essential for good bone, muscle and teeth development. The RDA of phosphorus for children and adults is 200 and 1000 mg respectively. Most of the treatments can supply more than 25% of RDA needed by adults. In this study, it was found that P content in roselle was not affected by parts of plants, cultivar and stages of growth. The interaction of cultivar with either plant parts or stages of growth also did not affect P content.

Zinc is vital for good brain development, bone formation and healing of wounds^34^. It is very important in nucleic acid synthesis. RDA of zinc is 4 and 14 mg for children and adults respectively^35^, most of these treatments can supply RDA for children. The Plant parts, stages of growth and their interactions with cultivar did not influence the content of Zn in roselle. The cultivar, however, influenced the level of Zn in roselle; red calyces higher than green calyces, red seeds also higher than green seeds.

Sodium is crucial for the maintenance of osmotic pressure in the body, maintenance of membrane potential and transmission of a nerve impulse. RDA for sodium is 1500 mg for an adult. Roselle dishes can be supplemented with exogenous Na to attain the RDA for adults. The values obtained in this study were, however higher than those reported by^19^ (0.9 – 2.9 mg) for roselle seeds and^27^ (12.5 mg) for calyces. Roselle calyces and leaves had higher Na than seeds in this study. The Na^+^/P^+^was however, lower than one for all the treatments. This indicates that the plants can be useful for a hypertensive patient if consumed. Noteworthy^36^, affirmed that for reducing cholesterol level, studies recommend 1,000 mg dried leaves, calyces or seeds 3 times daily; or 100 mg of standardized extract twice daily and also for hypertension 1 cup of roselle drink twice daily or dried powdered roselle extract of 250 mg per day was recommended.

This supports the reports of^37^ that affirmed roselle drink as an anti-hypertensive agent. Seeds contain lower Na than calyces and leaves^38^ found that Na content of calyces of green was higher than that of red. The interaction of plant parts and cultivar influenced the composition of Na in roselle, seeds having lower content. Red seeds were lower in Na than green while red calyces were higher in Na than green calyces. The interaction of the stage of growth and cultivar influenced the composition of Na value in roselle. The higher value recorded at pre-flowering stage of green cultivar while red cultivar highest at flowering stage and reduced at maturity of both cultivars. This is contrary to the report of^39^ who recorded an increase in Na content as growth continued.

Manganese is good for many functions in the body such as metabolism of amino acids cholesterol and glucose. It is an important element in blood formation, blood clotting and reduction of inflammation. In this study, it was found that Mn content in roselle was not affected by parts of plants, cultivar and stages of growth. The interaction of cultivar with either plant parts or stages of growth also did not affect Fe content.

Most of the mineral elements were found in significant proportion in both roselle cultivars and could be recommended for daily consumption. The consumption of vegetables such as roselle can supply important minerals in adequate quantity and can enhance one activity or the others in human physiology and helps in preventing chronic diseases^28^.

Vitamin A promotes resistance to diseases, delay in ageing and improve the health of eyes, nails and hairs^40^. The study showed that either leaves, calyces or leaves at any age of either red or green roselle can supply same content of vitamin A whereas interaction of both plant parts and cultivar influenced vitamin A content in roselle. Therefore, red roselle leaves were a better source of vitamin A while green roselle seeds and calyces were a better source of vitamin A than red. The value of absorbance of retinol (standard) which was lower than absorbance values at the pre-flowering stage of both cultivars may suggest that roselle at the vegetative stage are a better source of vitamin A (1236.15 and1228 µg retinol/100 g DW for green and red type respectively) having twice the RDI value (µ600) (FDA/WHO, 2012) and within the safe limit for consumption.

Vitamin C helps in building immunity against diseases, purification of blood and teeth and bones formation. Vitamin C content in calyces, which was observed to be the highest among the treatments (4.47 and 4.49 mg ascorbic acid/100 g DW) confirm the findings of Amin et al., (2006) that roselle drink contains higher vitamin C value than *Citrus sinensis*^41^ reported that antibacterial and antifungal properties of roselle was higher in calyces than leaves and seeds. This may be attributed to high vitamin C content in calyces of both cultivars. The study, however, showed that either leaves, calyces or leaves at any age of either red or green roselle can supply same content of vitamin A whereas interaction of both plant parts and cultivar influenced vitamin C content in roselle. Red leaves and seeds a better source of vitamin C than green leaves and seeds. Ascorbic acid (standard) absorbance value which was lower than all the treatments may suggest that any part of green or red roselle at any age contains higher vitamin C content than the popular ascorbic acid. This corroborates with the report^42^.

Vitamin E has been reported to be a good antioxidant necessary for the formation of red blood cells and also useful for recovery and maintenance of muscle and other tissues^43^. It was found that either seeds, calyces or leaves at any stage of growth of green or red roselle can supply the same content of Vitamin E. The interaction of plant parts and stages of growth with cultivar had no effects on vitamins E content in roselle. In other words, either leaves, calyces or seeds of green and red cultivars of roselle at any age (young or mature) gave the same content of vitamin E. Although the content of vitamin E in the tocopherol (standard) was recorded to be higher than the content in all the treatments, all the treatments contained vitamin E greater than 3X RDI for adult (10 mg alpha tocopherol/100 g)^33^.

Anti-nutrients are chemical compounds that reduce the nutrients utilization of food substances. Anti-nutrients can also be beneficial by mediating their effects on the human body through the same processes as conventional medicines^44^.

At the moderate level, phytate has a high affinity to bind zinc and lower the ratio of plasma zinc to copper and therefore lowers the risks of cardiovascular diseases^45^. At high dosage, it has been reported to reduce the availability of nutrients such as Ca and cause growth inhibition. However, subjecting the plants to processes such as soaking, boiling and cooking eliminates excess anti-nutrient sufficiently and thus rendering roselle good for consumption.

Saponin also reduces the availability of Ca in the body and causes various growth problems. However, if this anti-nutrient is present in very small amount, it is good for body physiology. Saponin has been shown to reduce blood glucose and insulin responses to starchy foods and/ or plasma cholesterol and triglycerides. Saponin below 10% in the foods is not hazardous to the body^46^. Green roselle had the highest saponin content at the flowering stage but the content reduced at post-flowering stage making it less harmful to consumers of mature roselle leaves. Nevertheless, all the treatments had saponin content acceptable for consumption.

Oxalate in the body at a small proportion is useful in reducing the risks of cancer. High content of oxalate in the body decreases the absorption of Ca and increase the formation of kidney stone^47^. Strong bonds are formed between oxalic acid with various other mineral elements such as Ca, Na, Mg and K to form oxalate. Ca oxalate in high amount can cause kidney stone. The concentration of oxalate in all the treatments (in the range of 0.22 – 0.92%) was within the safe limit.

Alkaloids are useful when present in small quantity, for instance, morphine is used as an analgesic, quinine as antipyretic and anti-malarial, reserpine as antihypertensive, however, high amount of alkaloid can be poisonous. Alkaloid content in all the treatments was within the safe limit (1.40 – 3.90%).

Anti-nutrient content in the two cultivars was within the safe limit and may be useful for the body^33^. The study indicated that alkaloid, oxalate, phytate and saponin contents in roselle were not influenced by plant parts (leaves, calyces and seeds) age of the plant and cultivar. This suggests that the content of alkaloid, oxalate, phytate and saponin present in the calyces is the same in seeds and leaves of green or red roselle of any age. In addition, the interaction of plant parts or stages of harvest with cultivars did not affect the anti-nutrient content of roselle. This report suggests that calyces, seeds and leaves (whether young or mature) of green or red roselle contains the same anti-nutrient content. This is in contradiction with the reports of^22^ who reported that leaves of *Amaranthus cruentus* at different ages contained different phytate, oxalate, alkaloid and saponin contents.

## Materials and methods

### Sample collection

Green and red cultivars of roselle were grown at the Medicinal Plant and Economic Development Research Center (MPED) experimental farm, Crop production farm, the University of Fort Hare, located at 32∘46′47′′ S and 26∘50′5′′ E and altitude of 524 m at East Alice in the Eastern Cape South Africa. Seeds of roselle were obtained from the Institute of Agricultural Research and Training (IAR&T) Ibadan, Nigeria. The identity of the seeds was confirmed in the herbarium of the institute.

Roselle leaves were harvested at pre-flowering, flowering and post-flowering stages of growth while calyces and seeds were harvested when mature. These were washed with water, chopped into smaller pieces, oven-dried at 40 °C until constant weights were obtained. The dried samples were then blended into a powdery form. The samples and the following analyses were managed and carried out under the same conditions for homogeneity. All the samples tested were in triplicates.

### Determination of proximate compositions

#### Determination of moisture content

The gravimetric method was used to determine the moisture content^47^. An empty porcelain crucible which was oven-dried at 105 °C for 1 h and cooled in desiccator was weighed (W_1_). Two gram (W_2_) of the dry sample was put into the crucible and oven-dried at 105 °C, cooled in the desiccator and weighed (W3), drying was repeated until a constant weight was achieved.

$$ {\text{Moisture}}\;{\text{content}} = \frac{{W_{2} - W_{1} }}{{W_{2} - W_{1} }} \times 100. $$

### Determination of total ash content

The procedure of^47,48^ were used to determine the ash content. A heat resistant crucible was oven-dried at 105 °C for 1 h, cooled and weighed (W_1_). Then 2 g of the sample was put in a weighed crucible and re-weighed (W_2_), ashed at 250 °C for 1 h and then incinerated at 600 °C for 5 h in a muffle furnace. This was later cooled in a desiccator and then weighed (W3). The % ash was calculated as:

$$\mathrm{\% Ash content}=\frac{W_{2}-W_{1}}{\mathrm{W}_{2}-\mathrm{W}_{1}}\times 100.$$

#### Determination of crude protein

Crude protein was determined by the Kjeldahl method as described by^47^. Two grams of the sample was digested in a Kjeldahl digestion tube and boiled with 20 ml of concentrated sulphuric acid and a catalyst until the mixture was clear. The digested sample was filtered and put into 250 ml volumetric flask distilled water was added to make up to 250 ml. The aliquot (50 ml of 45% sodium hydroxide solution) was transferred into a 500 ml round bottom flask and distilled. A flask containing 100 ml 0.1NHCL was used to collect 150 ml of the distilled and then titrated against 20 ml NaOH. Methyl orange was used as the indicator and change of colour to yellow indicates the endpoint.

Nitrogen content (%) was calculated using the following formula:

$$ \frac{{\left[ {\left( {{\text{ml standard acid }} \times {\text{ N of acid}}} \right) \, - \, \left( {{\text{ml blank }} \times {\text{ N of base}}} \right)} \right] \, - \, \left( {{\text{ml std base }} \times {\text{ N of base}}} \right) \, \times { 1}.{4}00{7} }}{{\text{Weight of sample in grams}}} $$

where; N is the normality, Crude protein (%) was obtained by multiplying the nitrogen value by a factor of 6.25, % crude protein = nitrogen in sample × 6.25.

#### Determination of crude fat

Five grams of the pulverized sample was extracted in 100 ml of diethyl ether by using an orbital shaker for 24 h. The extract was filtered and the ether extract was collected in a weighed beaker (W_1_). This was then equilibrated with 100 ml diethyl ether and shaken again for 24 h. The same beaker was used to collect the filtrate (W_1_). The content (ether) was concentrated to dryness in a steam bath and later dried in an oven at 40–60 °C. The beaker was then reweighed (W_2_). The crude fat content was calculated using the following formula:

$$\mathrm{\% Crude fat}=\frac{W_{2}-W_{1}}{\mathrm{Weight of the original sample}}\times 100.$$

#### Crude fibre determination

This was determined as described by^49^ with little modification. A glass crucible containing 2 g of the sample and attached to the extraction unit, boiling 1.25% sulphuric acid and solution (150 ml) was then added. The sample was digested for 30 min, after which the acid was drained out, and boiling distilled water was used to rinse the residue four times. The residue was then rinsed using 100 ml of 1.25% NaOH solution. Finally, the crucible was removed from the extraction unit and oven-dried at 110 °C (Overnight), allowed to cool in a desiccator and weighed (W_1_). Ashing of the sample was then done in a muffle furnace at 550 °C for five hours cooled in a desiccator and reweighed (W_2_).

The percentage of crude fibre was calculated using the following formula:

$$ = {\text{ Digested sample }}\left( {{\text{W}}_{{1}} } \right) \, {-}{\text{ Ashed sample }}\left( {{\text{W}}_{{2}} } \right)/{\text{ weight of the original sample }} \times {1}00 $$

$$\mathrm{\% Crude Fibre}=\mathrm{\% crude fibre}\frac{W_{1}-W_{2}}{\mathrm{Weight of the original sample}}\times 100.$$

#### Determination of carbohydrate (nitrogen free extract)

Subtraction of total protein, moisture content, crude fibre, ash and crude fat from the total dry matter (100) gives the carbohydrate content as follow:

$$ \% {\text{ Total carbohydrate }} = { 1}00 \, {-} \, (\% {\text{ moisture content }} + \, \% {\text{total ash }} + {\text{5 crude fat }} + \% {\text{ crude fiber }} + \% {\text{ crude protein)}}. $$

#### Determination of energy content

Energy value in Kilojoule per 100 g was calculated through the addition of the multiplied value for total carbohydrate, protein and crude lipid (ether extract), using the factor (16.736 kJ, 16.736 kJ and 37.656 kJ respectively) as follows:

$$ {\text{Energy value }}\left( {{\text{KJ}}/{1}00{\text{ g}}} \right) \, = \, \{ \left( {{\text{total carbohydrate }} \times {16}.{736}} \right) \, + \, \left( {{\text{crude protein }} \times {16}.{736}} \right) \, + \, \left( {{\text{crude fat }} \times { 37}.{656}} \right)\} . $$

### Determination of elemental composition

The elemental composition of the sample was determined by the use of 1 g of each treatment sample according to the method described by^50^ with the use of Inductively Coupled Plasma—Optical Emission Spectrometer (ICP- OES), Varian 710- ES Series, SMM Instruments, South Africa.

### Determination of vitamins content

#### Vitamin A

One gram each of pulverised samples of the plant was mixed with 20 ml of petroleum ether. This was poured out into a test tube and then left to evaporate to dryness. Later, 0.2 ml of chloroform–acetic anhydride (1:1, v/v) was added to the residue and 2 ml of TCA-chloroform (1:1, v/v) was added to the solution obtained. Vitamin A standard (retinol) was prepared using the same procedure for the samples. Final concentration of 1 mg/ml was made for both standard and plant extracts and serial dilution of the concentration was made given concentration levels 1, 0.8, 0.6, 0.4 and 0.2 mg/ml. The absorbance of the resulting solution and vitamin A standard was measured at 620 nm. The concentration of vitamin A in the sample was deduced from the standard curve. The vitamin A content is measured (in milligrams) as retinol activity equivalence/100 g (RAE/100 g DW).

#### Vitamin C

Vitamin C content of the samples was evaluated by pouring 20 ml of 0.4% oxalic acid into each of the samples (1 g each). The mixture was filtered and indophenol reagent was reacted with the filtrate in the ratio of 9 to 1 (filtrate: indophenol). Vitamin C standard (ascorbic acid) was prepared using the same procedure for the samples. The final concentration of 1 mg/ml was made for both standard and samples. Serial dilution of the concentration was later done giving concentration levels 1, 0.8, 0.6, 0.4 and 0.2 mg/ml. The absorbance of the resulting sample solution and standard was read at 520 nm. Vitamin C content in milligram ascorbic acid/100 g DW of the each of the samples was extrapolated from the standard curve.

#### Vitamin E

Vitamin E content was determined by mixing 1 g each of the samples with 20 ml ethanol. The mixture was filtered and to 1 ml of the filtrate, 1 ml each of 0.5% α-α-dipyridine and 0.2% ferric chloride in ethanol was added. The solution was made up to 5 ml with distilled water. Vitamin E standard (tocopherol) solution was also prepared using the same procedure as samples. The final concentration of 1 mg/ml was made for both standard and samples. Serial dilution of the concentration was later done giving concentration levels 1, 0.8, 0.6, 0.4 and 0.2 mg/ml. The absorbance of both samples solution and Vitamin E standard solution was read. Vitamin E content in milligrams alpha tocopherol/100 g DW was deduced from the standard curve.

### Determination of anti-nutrient content

#### Determination of saponin

The method described by^51^ was used with modification. One gram of the sample was soaked in 20 ml of 20% ethanol, heated in a water bath at 55 °C for 4 h, and stirred well. The content was filtered and re-extracted with 20 ml of 20% ethanol and shaken vigorously (not heated). This was filtered and the filtrate was added with the first filtrate. The filtrate was then reduced to one-quarter of its value in the water bath at 90 °C. This content was later poured into a separating funnel and 20 ml of diethyl ether was added into it. The content was shaken vigorously and attach back to the tripod stand. Aqueous / lower layer of the content was collected and ether/ upper portion containing fatty substances was discarded. The aqueous layer was then poured back into the funnel, and n- butanol was added and then shaken vigorously. A clean pre-weighed beaker was used to collect the butanol layer in the weighed beakers and evaporated to dryness. The residue was later dried in an oven until a constant weight is obtained.

$$\mathrm{\% Saponin content }= \frac{\mathrm{weight of residue}}{\mathrm{weight of the original sample}}\times 100$$

$$ \% {\text{ Saponin }} = {\text{ weight of residue }}\left( {\text{final sample}} \right)/{\text{weight of original sample }} \times {1}00. $$

#### Determination of phytate

Phytate content was determined as described by^52^ with modification. Sample of I g of the sample was put into a flask, 50 ml of 2% HCL was added and soaked for 3 h and then filtered. The filtrate (25 ml) was poured into another 250 ml conical flask and thiocyanate solution was added as an indicator. To attain the normal acidity, 53.5 ml of distilled water was added. The solution was then titrated against the standard Iron 111 chloride solution (0.00195 g of iron per ml) until a brownish yellow colour persisted for 5 min.

Phytate was calculated as follows:

$$ {\text{Phytate }}\left( \% \right) \, = {\text{ Titre value }} \times 0.00{195 } \times {1}.{19 } \times {1}00. $$

#### Determination of oxalate content

The method described by Aina et al. (2012) and modified by Unuofin et al. (2017) was adopted to determine the content of oxalate in the sample. Briefly, 1 g of the ground sample was poured into a conical flask, 75 ml of 3 M sulphuric acid was added and mixed well for an hour using a magnetic stirrer. The solution was filtered and 25 ml of the filtrate was collected and heated to 85 °C and kept at a temperature above 70 °C throughout the experiment. The hot aliquot of the filtrate was titrated against 0.05 M/l of KMnO4 until the light pink colour, which persists for 15 s, was reached; this is the endpoint of the reaction. The oxalate content was calculated by taking 1 ml of 0.05 M/l of KMnO4 as equivalent to 2.2 mg oxalate.

#### Alkaloids

The method of^53^ was adopted to determine the alkaloid content in the samples. Acetic acid (10%) was prepared in ethanol and 1 g of the sample was soaked in 50 ml of 10% acetic acid for 4 h. The content was later filtered using a vacuum pump and concentrated in a water bath to a small quantity. Concentrated Ammonium hydroxide was added dropwise until precipitate (until the cloudy fumes cease). The solution was allowed to cool down, then filtered using the pre-weighed filter paper. The filter paper retained the precipitate/ residue. The precipitate on the filter paper was oven-dried and weighed.

$$ \% \, = {\text{ weight of the precipitate}}/{\text{weight of the sample}} $$

$$\mathrm{\% alkaloid }=\frac{\mathrm{weight of precipitate}}{\mathrm{weight of the original sample}} \times 100$$

### Statistical analysis

All experiments were done in triplicates and the results expressed as Mean ± SD using the Microsoft excel 2010 spreadsheet. Data were analysed using ANOVA. Means were segregated using Tukey paired wise comparison. The means were treated as significantly different at p < 0.05.

## Conclusion

Plant parts, stages of growth and cultivars of roselle had no influence on nutritional contents of roselle except (i) crude fat where green roselle had higher value at maturity while red was high at young age; (ii) Moisture where green was more than that in red and (iii) Ash where red calyces had higher content than green calyces. The interaction of plant parts and cultivars did not influence the nutritional composition of roselle apart from crude fat content in seeds which was higher than leaves and calyces. Interaction of stages of harvest and cultivar had no contribution to the nutritional level of roselle except (i) carbohydrate which was higher in red roselle leaves at vegetative stage and green at maturity and (ii) energy which was lower than green during flowering and vice versa at post flowering stage.

Similarly, the factors did not influence the elemental composition of roselle except (i) Na which was higher in calyces and leaves than seeds and red seeds; (ii) Zinc where red calyces and seeds were higher than green; (iii) Copper where red seeds had higher content than green, and red leaves higher than green at vegetative stage. Interaction of plant parts and cultivar had no influence on the nutritional composition except magnesium whose content was lower in seeds than other parts, and also had higher content in red seeds than green seeds while green calyces higher than red calyces and (ii) Sodium where red seeds were lower than green seeds and red calyces higher than green calyces. The interaction of stages of growth and cultivars had no effect on mineral content of roselle except Na content which was higher in green leaves at a vegetative stage and red had higher at maturity.

Moreover, all the parameters had no effect on vitamins. The interaction of plant parts with cultivars, however, contributed to higher vitamin A content in the leaves of red roselle than green roselle while green seeds and calyces were better sources of vitamin A than red. The interaction also influenced Vitamin C value where the leaves and seeds were better source of vitamin C than green leaves and seeds. None of the factors influenced anti-nutrient content.

## Data Availability

Data were presented in the manuscript.
